# A modified animal model of hepatic regeneration induced by hilar bile duct ligation

**DOI:** 10.1038/s41598-021-99758-z

**Published:** 2021-10-12

**Authors:** Tao Li, Yichao Chai, Pengkang Chang, Fenggang Reng, Zhao Xue, Hongke Zhang, Yi Lv, Liangshuo Hu

**Affiliations:** 1 Department of Hepatobiliary Surgery and Institute of Advanced Surgical Technology and Engineering, The First Affiliated Hospital of Xi’an Jiaotong University, Xi’an, China; 2 Department of Oncology, The Second Affiliated Hospital of Xi’an Jiaotong University, Xi’an, China

**Keywords:** Experimental models of disease, Cell proliferation, Disease model

## Abstract

Mechanisms of the proliferation of liver are mainly studied in animal model of liver regeneration after partial hepatectomy (PH). However, the PH model involves complex regeneration mechanisms, including hemodynamic factors, cytokines, growth factors, and metabolites. Among liver metabolites, bile acid (BA) is a key signaling molecule that regulates liver regeneration. This study aimed to establish a new type of rapid liver hyperplasia model induced mainly by bile acid pathway through hepatoenteral circulation with hilar bile duct ligation (HBDL). We first established the HBDL model by ligating the bile duct of all hepatic lobes but the right lateral lobe in rabbits and compared with the PVL model and sham operation group. Changes in the liver lobe and hemodynamics were observed. Liver function and the bile acid level were also analyzed. Then we verified the HBDL model in mice. Liver function and the levels of bile acids and cytokines were tested. The protein and mRNA levels of FXR, FGF15, CYP7A1 and FoxM1b in liver tissue were also analyzed. After hilar ligation of the biliary tract, the unligated liver lobes proliferated significantly. Compared with those in the sham group, the volume and weight of the unligated right lateral lobe of the liver in the HBDL group and the PVL group increased significantly (*P* < 0.05). Transient liver function impairment occurred both in the HBDL group and PVL group in the rabbit model as well as the mouse models. The bile acid levels in the HBDL groups of the rabbit model and mouse model increased significantly within first week after surgery (*P* < 0.05). The immunohistochemistry results confirmed the proliferation of hepatocytes in the unligated liver lobe. Compared with those in the sham group, the levels of FXR, FGF15 and FoxM1b in the HBDL group were significantly increased (*P* < 0.05), while the expression of CYP7A1 was inhibited. Compared with those in the HBDL group, the postoperative hemodynamic changes in the PVL group were significant (*P* < 0.05). The levels of IL-6 and TNF-α in the HBDL group were higher than those in the sham group. The HBDL model is simple to establish and exhibits good surgical tolerance. The model has definite proliferative effect and strong specificity of bile acid pathway. This is an ideal animal model to study the mechanism of liver regeneration through bile acid pathway.

## Introduction

The liver has tremendous regenerative potential than any other tissue in adult organisms^1^. In the past decades, information on the mechanisms related to the proliferation of liver cells and tissues has mainly been studied through experimental models of hepatic regeneration after partial hepatectomy (PH)^2–4^. However, hepatic regeneration entails the activation of multiple regulatory pathways. In addition, the nature of the molecules and signals that participate in the regenerative response is diverse^2^. Therefore, research on the mechanism of hepatic regeneration needs to be more detailed and precise.

Recent studies have shown that among liver metabolites, bile acid (BA) is considered to be a key signaling molecule that regulates hepatic regeneration^5^. It has been observed that during hepatic regeneration after PH in mice, circulating and intrahepatic levels of bile acids (BAs) fluctuate. Moreover, BA signaling is related to the initiation of the hepatoproliferative response in rodents and liver growth after portal vein embolization in humans^6–9^. An experimental study showed that serum bile acid levels increased during regeneration in mice after PH. Feeding wild-type mice a diet supplemented with nontoxic concentrations of bile acids (0.2% cholic acid) increased the volume of the intact liver by 30% and increased DNA synthesis. This treatment also enhanced hepatic regeneration in a rodent model of 70% PH. Conversely, feeding mice a bile acid chelating resin (cholesteramine) that reduces bile acid levels significantly delayed hepatic regeneration after PH^10,11^. Several experimental studies have shown that the maintenance of serum bile acid levels and enterohepatic circulation are essential for maintaining normal hepatic regeneration^12,13^. When the enterohepatic circulation of BAs is interrupted, hepatic regeneration is significantly inhibited^14^. BAs can initiate and promote hepatocyte proliferation by activating the nuclear BA receptor FXR^15^. In addition, after PH, mice deficient in the ileal secretory intestinal factor FGF15 (a downstream molecule of BA) showed evidence of delayed hepatic regeneration, weakened activation of signaling pathways involved in hepatic regeneration and decreased expression of genes that drive the cell cycle (such as FoxM1b)^16,17^. In summary, this evidence strongly supports the close relationship between bile acids and hepatic regeneration and indicates that there are complex mechanisms that specifically regulate BA levels and circulation during this process. Therefore, BA is increasingly recognized as a key player that regulates hepatic regeneration^16^, and the related mechanisms by which BAs induce liver proliferation are being actively explored.

The current research on hepatic regeneration mostly uses the animal model of partial hepatectomy established by Higgins et al. in 1931; in this model, the left and middle lobes of the rat liver are excised, and the excised volume accounts for approximately 60% to 70% of the total liver volume^18^. In addition, animal models of liver leaf hyperplasia induced by portal vein embolism were also established^19^. However, animal models of hepatic regeneration induced by PH or portal vein embolism involve many regenerative mechanisms^1^, such as hemodynamic factors, cytokines, growth factors, and metabolites^2,20^, which increases the complexity of research on regeneration-related mechanisms ^21^. Therefore, research on the effect of BAs on hepatic regeneration and the related mechanisms still lacks a highly targeted model with a rapid proliferation rate. Bile duct ligation (BDL) can cause significant increases in bile acid levels in the body^22–24^, but this method causes severe liver damage in mice^25,26^. In this study, hilar bile duct ligation (HBDL) was used to establish a new type of rapid hepatic hyperplasia induced by enterohepatic circulation of bile acid that is highly targeted and highly reproducible.

## Method

### Animals

Eighteen healthy male New Zealand rabbits weighing 2.5–3.0 kg were purchased from the Experimental Animal Center of Xi'an Jiao Tong University. A total of 30 male C57BL/6 mice were purchased from the Experimental Animal Center of Xi'an Jiao Tong University (8–10 weeks old, weighing 34–49 g, with an average weight of 39.9 ± 4.8 g). Before the experiment, the rabbits and mice were given a standard laboratory diet. Water and food were freely available, and a 12-h circadian cycle was maintained under constant environmental conditions. The animal experiment protocol of this paper was approved by the Laboratory Animal Administration Committee of Xi'an Jiaotong University (approval No. XJTULAC 2020–1374) and carried out in accordance with the animal experiment regulations of Xi'an Jiaotong University and the basic guidelines for animal experiments. The study is reported in accordance with ARRIVE guidelines.

### Animal models and surgery

The rabbits were divided into three groups: the HBDL group, PVL group and sham group. In the hilar bile duct ligation (HBDL) group (6 animals), after intravenous anesthesia with 3% pentobarbital sodium 30 mg/kg, a midline incision in the upper abdomen was made to expose the hepatic hilum. The bile ducts of each lobe were exposed, and the right lateral lobe bile duct and common bile duct were left unobstructed. The remaining bile ducts of the liver lobes were ligated with silk thread. The abdomen was then closed. In the portal vein ligation (PVL) group (6 animals), after anesthesia, a midline incision was made in the upper abdomen to expose the hepatic portal, and the portal vein and branches were accurately dissected. The branch of the right lateral lobe portal vein and the main trunk of the portal vein were retained. The remaining liver lobe portal vein branches were ligated with silk thread, and the abdomen was closed with a continuous suture. In the sham group (6 animals), laparotomy was performed after anesthesia, the hepatic portal was exposed, and the abdomen was closed.

The mice were divided into two groups: the HBDL group and the sham group. In the HBDL group (15 animals), the animals were anesthetized by inhalation with an animal anesthesia instrument; the concentration of isoflurane was 3%, and the flow rate was 0.5 L min^−1^. After successful anesthesia, a mid-incision in the upper abdomen of the mouse was opened to expose the hepatic hilum. The bile ducts of each lobe were exposed, and the caudate lobe bile duct and common bile duct were left unobstructed. The remaining hepatic bile ducts were ligated with silk thread, and the abdomen was sutured continuously to close the incision. In the sham group (15 animals), laparotomy was performed after anesthesia, the hepatic portal was exposed, and the abdomen was closed. Please refer to the Supplementary Information file for the specific surgical methods used in this study (Supplementary Fig. S1 and Table S1). We have subsequently supplemented the experiment of the mouse model of partial hepatectomy and compared it with the HBDL model.

After surgery, the animals were given free access to a regular diet and water. All animal operations were performed by the same surgeon under sterile conditions. After surgery, the animals recovered on a heating pad, and rabbits received buprenorphine (0.05 mg/kg; iv) for pain relief every 24 h, up to 2 days after surgery. Because the surgical incision was a Class I incision, no antibiotics were used. After completing the experiment, the rabbits were euthanized under anesthesia [ketamine (40 mg/kg) + xylazine (4 mg/kg); iv].

### Laboratory examination

Blood samples were collected from rabbits in each group before and the first day, the first week, and the second week after surgery. At each time point, approximately 3 to 5 ml of blood was drawn from each rabbit through the ear vein. The mice in each group were divided into two batches according to the time of blood collection. Each batch consisted of 18 and 12 animals. Approximately 0.5 ml of blood was collected from the orbit of each mouse the first day and first week after the operation, and the mice were sacrificed by cervical dislocation. Blood samples were centrifuged at 4 °C, centrifuged at 3000 r/min for 15 min, and the supernatant was used to measure AST (Aspartate aminotransferase), ALT (Alanine aminotransferase), TBIL (total bilirubin), ALP (alkaline phosphatase), GGT (γ-glutamyl transpeptidase) and ALB (albumin) levels with a Chemray 240 (Rayto). Total bile acid (TBA) was determined by Total Bile Acid (TBA) Determination Kit (E003-2, NanJing JianCheng Bioengineering Institute). The instruments used in the test include a miniature high-speed centrifuge (C2500-R-230 V, Labnet, America), an electric thermostatic incubator (ICV-450, ASONE, Japan), and FlexStation 3 multifunctional microplate (Flexstation3, Molecular Devices). The body weight, total liver weight, total liver volume, right lateral lobe weight and right lateral lobe volume of the rabbits were measured before the operation and at the first week and the second week after the operation. The volume of liver lobes was measured by drainage methods. The volume of hepatic lobe was reflected in the change of water level. A VlNNO 70 color Doppler ultrasound diagnostic instrument, with a high -frequency probe (L12—5) and a frequency of 5 ~ 12 MHz, was used before surgery, immediately after surgery, and the second week after surgery to measure the right lateral lobe, portal vein velocity, portal vein trunk and branch diameters, and hepatic artery velocity in the rabbits.

Volume A (VA) is the volume of the right lateral lobe liver measured before surgery; Volume B (VB) is the volume of the right lateral lobe liver measured the second week after surgery. The amount of liver regeneration was calculated as VB-VA, and the percentage of liver regeneration was calculated as 100% × (VB-VA)/VA.

### Histological and immunohistochemical staining

Pieces of liver were harvested from the mice. Sections of these pieces were stored in 4% neutral buffered formalin for 24-to-48 h and then embedded in paraffin. Three-micron-thick tissue sections of paraffin blocks were obtained from all animal groups and stained with hematoxylin–eosin (HE) and proliferating cell nuclear antigen (PCNA). After HE staining, the general liver morphology and histological changes caused by hilar biliary ligation or portal vein ligation were qualitatively evaluated. PCNA staining (Anti-PCNA Rabbit pAb, GB11010, Servicebio) was used to assess hepatocyte proliferation. The main reagents include Histochemical kit DAB chromogenic agent (G1211, Servicebio). The protein expression of PCNA is positive by the appearance of brown or brown particles in the nucleus. The number of PCNA-positive hepatocytes was determined in 20 random fields (400 ×) in different sections from each group, and the percentage (%) of PCNA-positive hepatocytes among total hepatocytes was calculated.

### ELISA

At the designated time points after surgery (the first day and the first week), serum was obtained from each group of mice. The serum concentrations of tumor necrosis factor-α (TNF-α) and interleukin 6 (IL-6)were measured using ELISA kits (Mouse TNF-α ELISA Kit, E-EL-M0049c, Elaret and Mouse INTERleukin-6 ELISA Kit, E-EL-M0044c, Elaret). The instruments used in the test include a miniature high-speed centrifuge (C2500-R-230 V, Labnet, America), an electric thermostatic incubator (ICV-450, ASONE, Japan), and FlexStation 3 multifunctional microplate (Flexstation3, Molecular Devices). Before the mice were sacrificed, portal vein blood was obtained. Using an enzyme-linked immunosorbent assay kit (for FGF15) (Huamei), the FGF15 protein level in mouse portal vein blood was measured.

### Western blot analysis

Total tissue protein was extracted. The BCA method was used for protein quantification. For electrophoresis, an SDS-PAGE gel was prepared, the protein sample (40 μg) and marker were added to the loading wells with a micropipette. Electrophoresis at a constant voltage of 80 V was performed for approximately 1.5 h. For electrotransfer, the gel was removed and placed in the instrument in the following order: black plate-fiber mat, filter paper, gel, PVDF membrane, filter paper, fiber mat, and white plate. After the plate was clamped, it was placed in the film transfer instrument, and the transfer was performed. The PVDF membrane was placed in TBST (blocking solution) containing 5% skimmed milk powder and shaken at room temperature for 2 h. For primary antibody incubation, the corresponding primary antibody was diluted with blocking solution, and the PVDF membrane was incubated in the primary antibody solution overnight at 4 °C. For secondary antibody incubation, the corresponding HRP-labeled secondary antibody was diluted with blocking solution, and the PVDF membrane was with in the secondary antibody for 2 h at 37 °C on a shaker. For color exposure, the enhancement solution in the ECL reagent was mixed with the stable peroxidase solution at a ratio of 1:1, the working solution was added dropwise to the PVDF membrane and incubated for several minutes, after which the film was developed, fixed, washed, and scanned sequentially. The gray value was analyzed with bandscan.

### mRNA expression analysis

mRNA expression was determined by real-time fluorescence quantitative PCR. Total RNA was extracted by the TRIzol method (Ambion). The purity and concentration of RNA were determined by a microspectrophotometer. Reverse transcription (RT) was performed to synthesize total RNA into cDNA. The reaction system for real-time fluorescence quantitative PCR analysis was as follows:

**Table:** 

cDNA	4 μl
Forward Primer (10 μM)	0.4 μl
Reverse Primer (10 μM)	0.4 μl
SYBR Green Master Mix(VAZYME)	10 μl
50 × ROX Reference Dye 2	0.4 μl
H_2_O	4.8 μl

Reaction procedure:

**Table:** 

Project	Temperature (° C)	Time	Number of cycles
Predenaturation	95	10 min	1
Denaturation	95	15 s	40
Annealing and extension	60	60 s	
Melting curve collection	95	15 s	1
	60	60 s	
	95	15 s	

The primer sequences were synthesized by Kinco.

**Table:** 

Gene	Primer	Sequence (5'-3')	PCR products
β-actin	Forward	CACGATGGAGGGGCCGGACTCATC	240 bp
	Reverse	TAAAGACCTCTATGCCAACACAGT	
Mus FXR	Forward	GCAGACCAACAGACCCTCCT	274 bp
	Reverse	TATTGAAAATCTCCGCCGAA	
Mus FoxM1b	Forward	CCCTGTCTGGATGAGCCTGA	148 bp
	Reverse	CCTTGATGGGGGTCTTGAAA	
Mus CYP7A1	Forward	GGCATTTGGACACAGAAGCA	196 bp
	Reverse	ATACATCCCTTCCGTGACCC	

SYBR Green dye was used to analyze relative quantitative PCR.

### Statistical analysis

All data were statistically analyzed with SPSS software, and the measurement data are shown as the mean ± standard deviation (SD). Using ANOVA, significant differences between multiple study groups were tested. When comparing only two groups, an unpaired t-test was used. A value of *P* < 0.05 was considered statistically significant.

## Results

### Liver hyperplasia after hilar bile duct ligation

**Figure 1:**
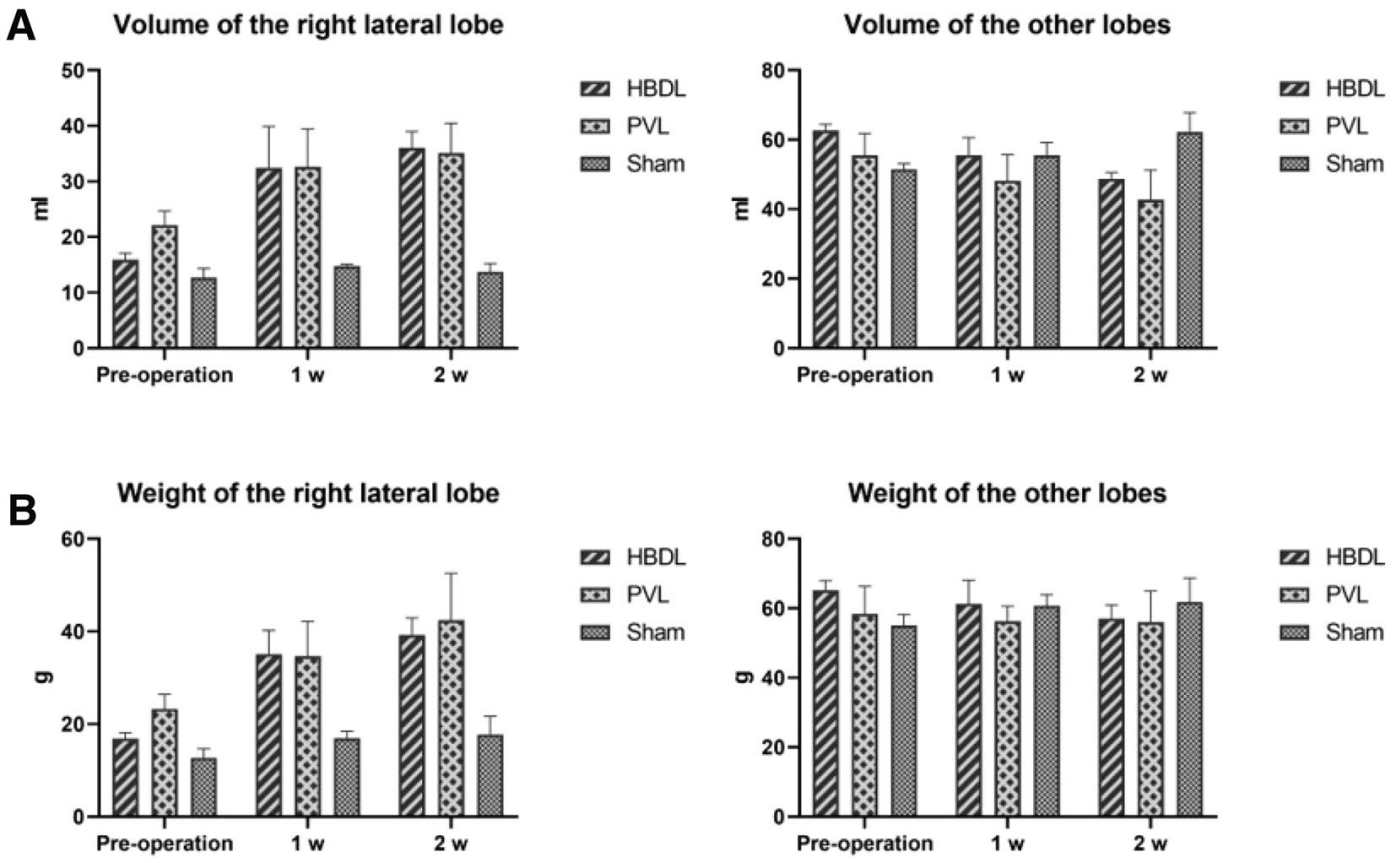
Changes in the volume of the right lateral lobe and the remaining liver lobes in the three groups of rabbits at different time points (**A**); changes in the weight of the right lateral lobe and the remaining liver lobes in the three groups of rabbits at different time points (**B**).

In this study, HBDL and PVL rabbit models were established, and changes in liver volume and weight were recorded, as shown in Fig. 1 (Supplementary Tables S2 and S3). Two weeks after the operation, compared with those in the sham operation group, the volume and weight of the unligated right lateral lobe of the liver in the HBDL group and the PVL group were significantly increased (*P* < 0.05). However, the volume and weight of the ligated liver decreased to some extent. The right lateral lobe volumes in the HBDL group and PVL group were 15.9 ml and 22.2 ml, accounting for 20.3% and 28.6% of the total liver volume, respectively. At 2 weeks after the operation, the right lateral lobe volumes in the HBDL group and PVL group were 34.9 ml and 36.9 ml, accounting for 38.6% and 43.4% of the total liver volume, respectively. At 2 weeks after the operation, the amount of liver regeneration in the HBDL group and PVL group was 19.0 and 14.7 ml, and the percentage of liver regeneration was 119.5% and 66.3%, respectively. There was no significant difference in liver regeneration between the two groups (*P* > 0.05). In the established mouse model, the liver also exhibited a similar proliferative response.

### Changes in liver function after hilar bile duct ligation

In the rabbit model, compared with the sham group, the HBDL group and PVL group had transient liver damage. As shown in Supplementary Fig. S2, in the HBDL group, AST and ALT levels increased significantly on the first day after surgery and were 548.5 ± 111.49U/L and 507.41 ± 106.86 U/L, respectively. Compared with that of the sham group, the difference was statistically significant (*P* < 0.05). AST and ALT gradually decreased, reaching 71.86 ± 34.92 U/L and 75.43 ± 24.50 U/L, respectively, in the second week after surgery. The changes in AST and ALT levels in the PVL group were similar to those of the HBDL group. The levels were significantly increased on the first day after surgery and were 787 ± 150.4 U/L and 587.25 ± 80.84 U/L, respectively. Compared with that of the sham group, the difference was statistically significant (*P* < 0.05). The degree of the increase was significantly higher than that of the HBDL group. However, at the first week after surgery, compared with those in the HBDL group, AST and ALT levels decreased significantly to 24.8 ± 9.93 U/L and 79 ± 34.81 U/L, respectively, and reached normal values at two weeks after surgery. The albumin level of the HBDL group decreased slightly after surgery, and there was no significant difference compared with that of the sham group (*P* > 0.05). There was no significant change in the albumin level in the PVL group after surgery, and there was no statistically significant difference compared with that of the HBDL group (*P* > 0.05). In the HBDL group, the ALP level increased significantly (293.5 ± 89.34 U/L) on the first day after surgery and was approximately 3 times that of the PVL group (*P* < 0.05). The ALP level gradually decreased and dropped to a normal level 2 weeks after surgery. The GGT level exhibited a significant increasing trend the first week after surgery (187.2 ± 67.45 U/L) and was significantly different from that of the PVL group (*P* < 0.05). Then, the level gradually decreased to 54.8 ± 40.39 U/L, which was still higher than normal. The level of bilirubin in the HBDL group was higher than that of the other two groups on the first day after surgery. There was no significant change in the level of bilirubin at the other time points in the three groups compared with that before surgery.

In the mouse model, as shown in Supplementary Fig. S3, the HBDL group had liver function impairment at the first week after surgery. The levels of AST, ALT and bilirubin were significantly higher than those in the sham group, and the difference was statistically significant (*P* < 0.05).

### Hilar bile duct ligation promotes liver hyperplasia through enterohepatic circulation of bile acid

**Figure 2:**
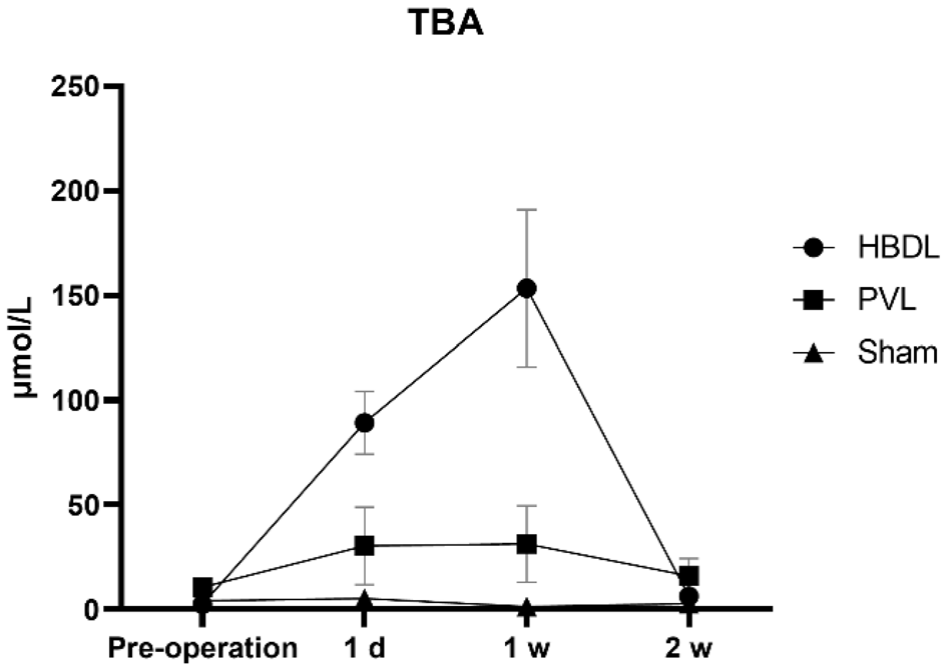
Serum bile acid levels in the three groups of rabbits at different time points.

We hypothesized that the enlargement of the unligated liver lobe after biliary tract ligation was due to the effects of bile acid, which induces rapid and continuous hepatocyte proliferation. Therefore, the total bile acid levels were measured in the rabbit and mouse models and the expression of receptor genes was verified in the mouse model. In the rabbit model, as shown in Fig. 2, the level of bile acid in the HBDL group was significantly increased in the first week after surgery, reaching 153.3 ± 26.5 µmol/L, which was approximately 5 times that of the PVL group (*P* < 0.05). Afterward, bile acid levels gradually decreases.

**Figure 3:**
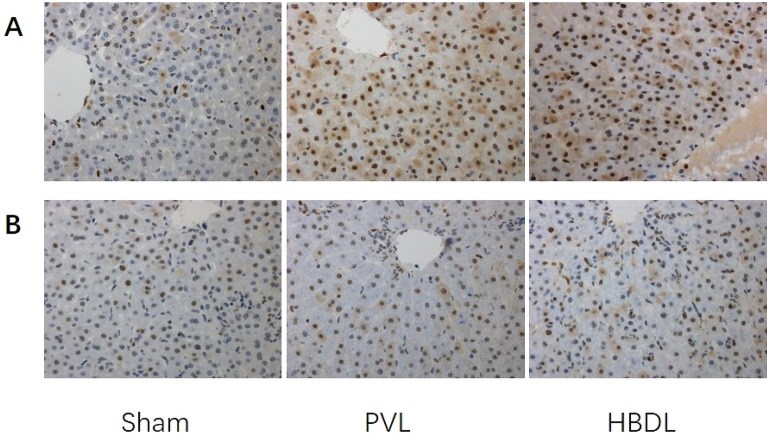
DNA synthesis in hepatocytes was assessed at different time points by immunohistochemical evaluation of PCNA incorporation into DNA. The first week after surgery (**A**); two weeks after surgery (**B**). (× 400).

PCNA staining showed that the ratios of positive cells in the HBDL and PVL groups were significantly higher than that in the sham group, and the difference was statistically significant (*P* < 0.05). The ratio of PCNA-positive cells in the HBDL group was approximately 55.4 ± 7.5% at the first week after surgery and 33.4 ± 5.6% at 2 weeks after surgery. Compared with that of the PVL group, the difference was not statistically significant (*P* > 0.05) (Fig. 3 and Supplementary Table S4).

**Figure 4:**
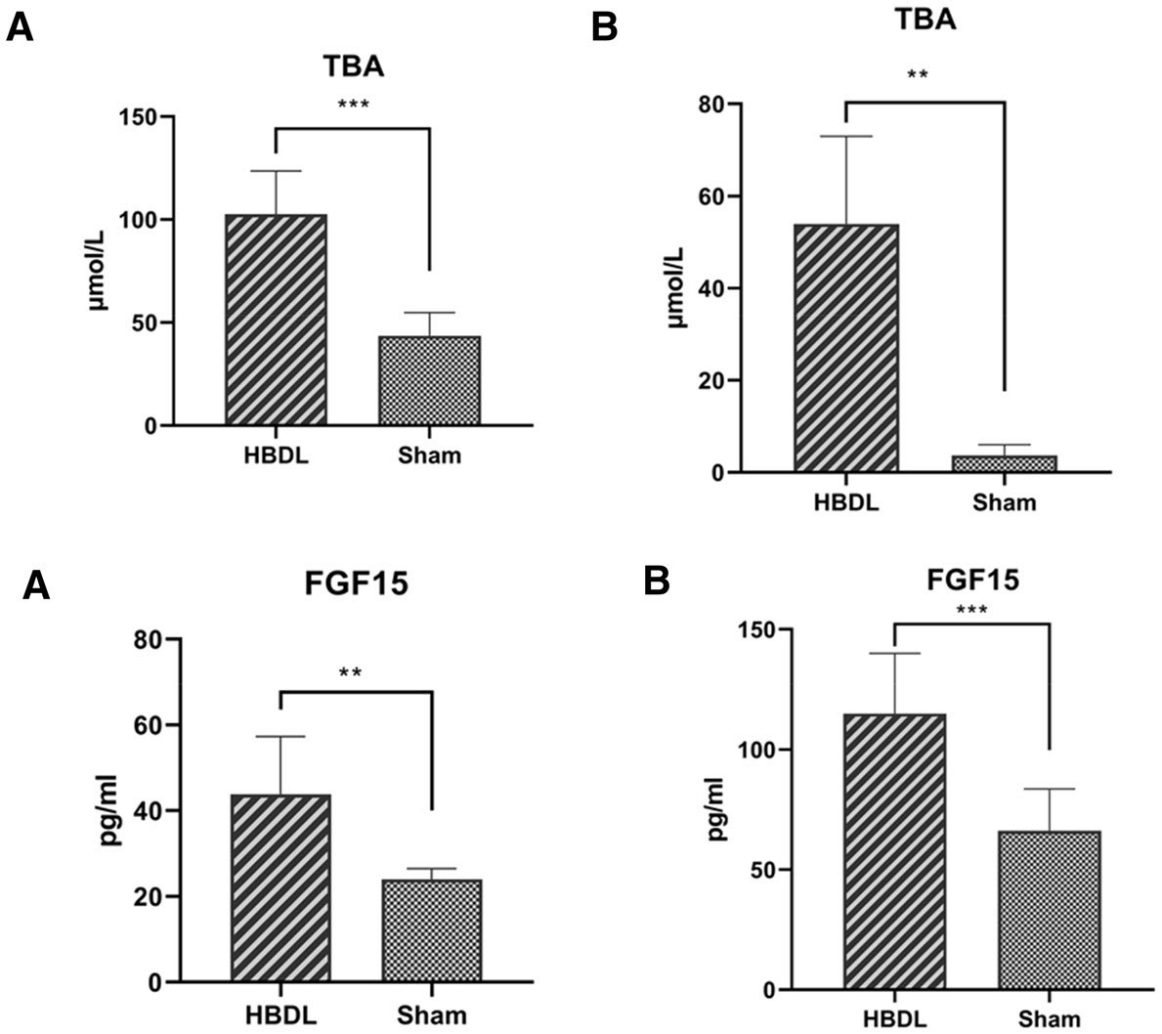
Serum levels of bile acid and FGF15 levels in the portal vein blood in HBDL mice at different time points, *p < 0.05 versus the sham group. The first day after surgery (**A**); the first week after surgery (**B**).

In the mouse model, the level of bile acid in the HBDL group was significantly increased the first day and the first week after the operation (Fig. 4), which was significantly different from that in the sham operation group (*P* < 0.05). Compared with that in the sham operation group, the level of FGF15 in portal vein blood in the HBDL group was significantly increased the first day and the first week after the operation (Fig. 4), and the difference was statistically significant (*P* < 0.05).

**Figure 5:**
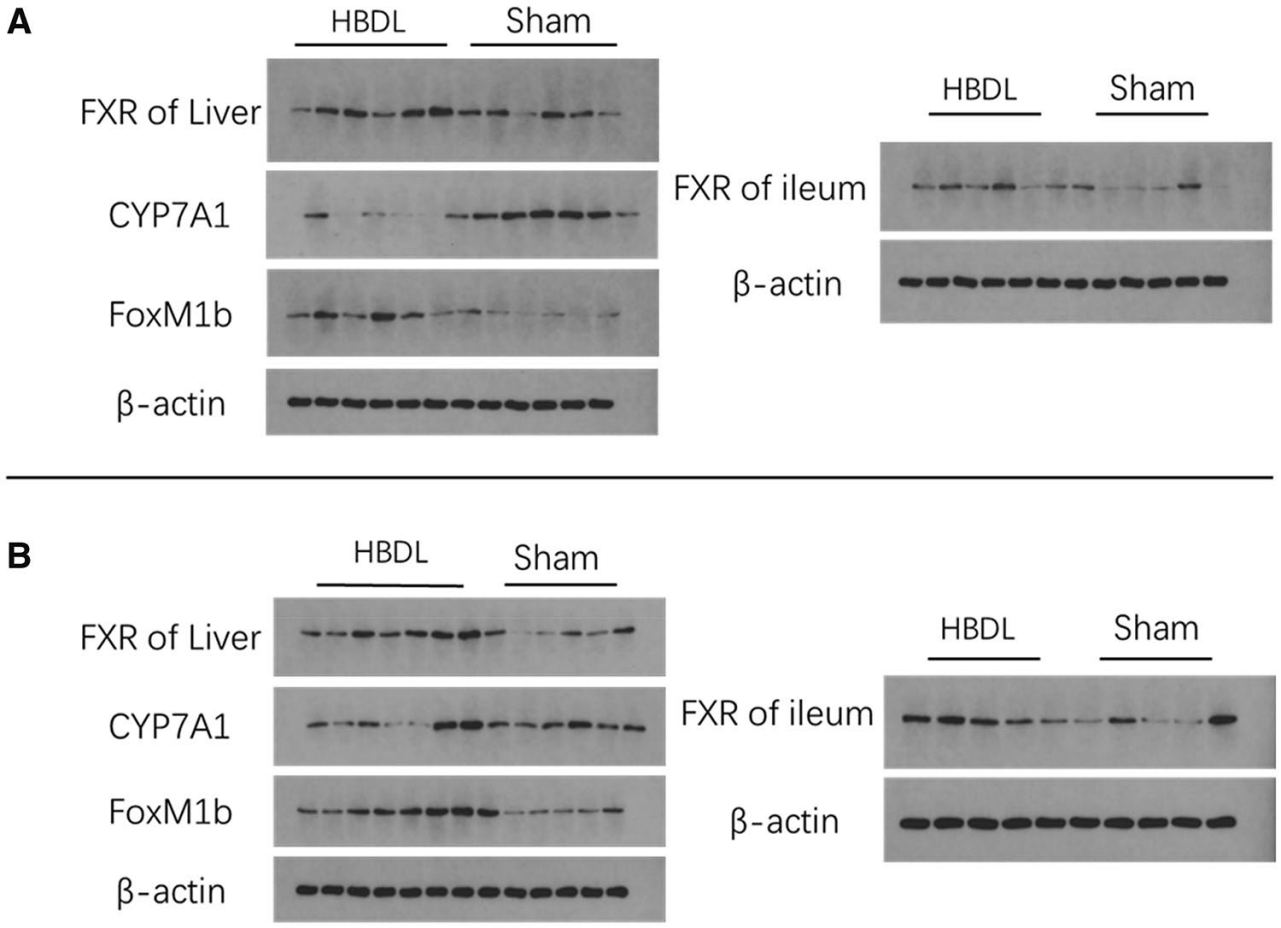
Expression of FXR in the livers, FXR in the ileum, CYP7A1 in the livers and FoxM1b in the livers of HBDL mice at the protein levels at different time points. Representative blots of HBDL (left side of membranes) and Sham (right side of membranes) at the first day after the operation (**A**) and the first week after the operation (**B**) is indicated.

**Figure 6:**
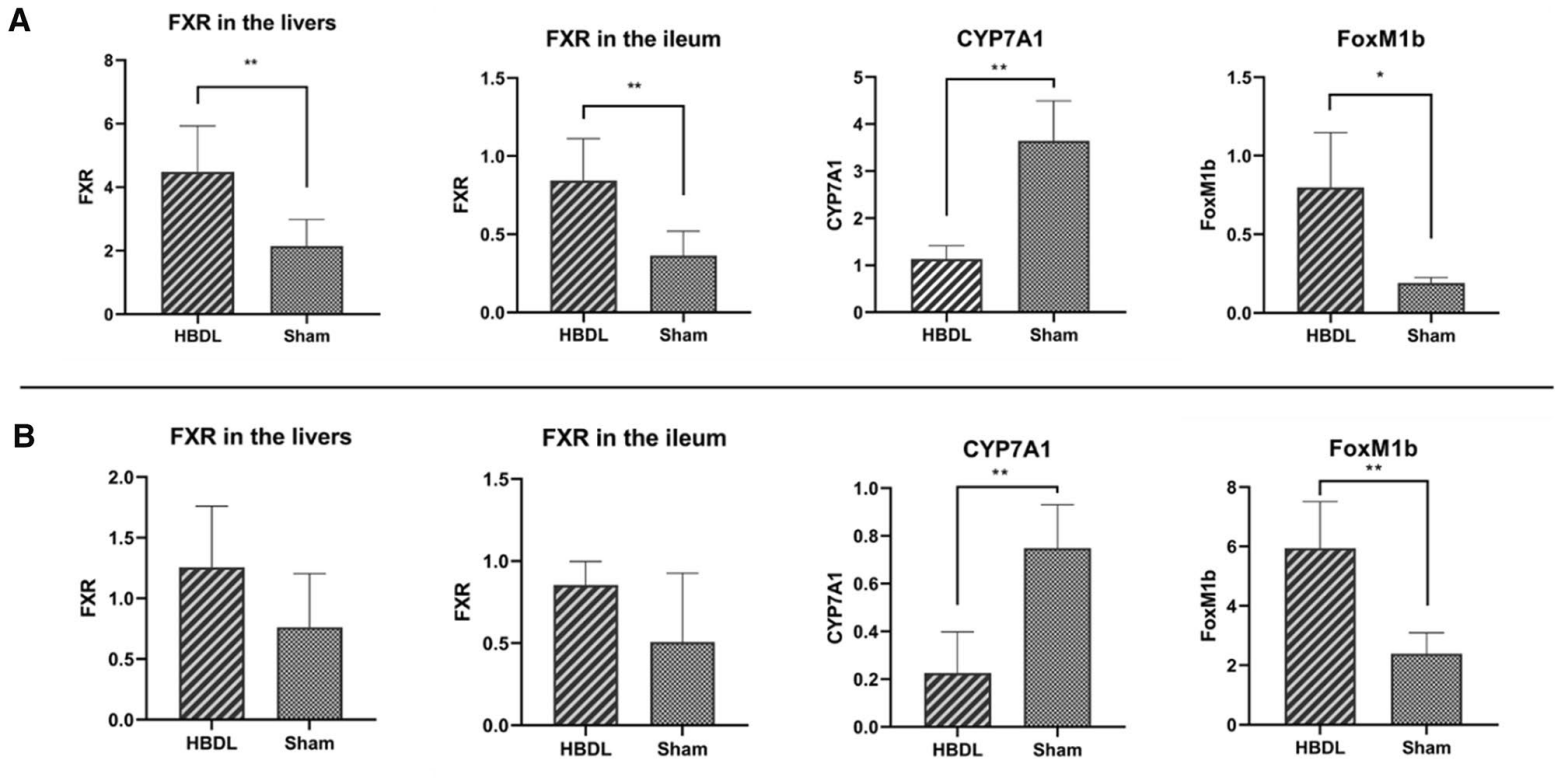
Expression of FXR in the livers, FXR in the ileum, CYP7A1 in the livers and FoxM1b in the livers of HBDL mice at the mRNA at different time points. The first day after the operation (**A**) and the first week after the operation (**B**) is indicated. *p < 0.05 versus the sham group.

We measured the mRNA and protein expression levels of the receptor gene in the liver and ileum of mice the first day and the first week after the operation. As shown in Figs. 5 and 6, the first day after the operation, the mRNA expression of farnesoid X receptor (FXR) in the liver and ileum in the HBDL group was significantly higher than that in the sham group (*P* < 0.05). The protein expression of FXR in the liver and ileum in the HBDL group was slightly higher than that in the sham group. One week after the operation, the FXR mRNA level in the HBDL group was still higher than that in the sham group, but the difference was not statistically significant (*P* > 0.05). FXR protein expression levels in the liver and ileum in the HBDL group were higher than those in the sham group, and the difference was statistically significant (*P* < 0.05). Cholesterol 7α-hydroxylase (CYP7A1) is a key rate-limiting enzyme that regulates the synthesis of BA. The mRNA expression of CYP7A1 in the HBDL group was significantly decreased the first day and the first week after the operation, which was statistically significant compared with that in the sham group (*P* < 0.05). The first day after the operation, CYP7A1 protein expression in the HBDL group was significantly lower than that in the sham group (P < 0.05). One week after the operation, CYP7A1 protein expression in the HBDL group was still lower than that in the sham group (Figs. 5 and 6). The first day and the first week after the operation, the mRNA and protein expression of FoxM1b in the HBDL group was significantly increased compared with that in the sham group (*P* < 0.05) (Figs. 5 and 6). (Supplementary Figs. S4 and S5).

### Changes in hemodynamics and cytokines

**Figure 7:**
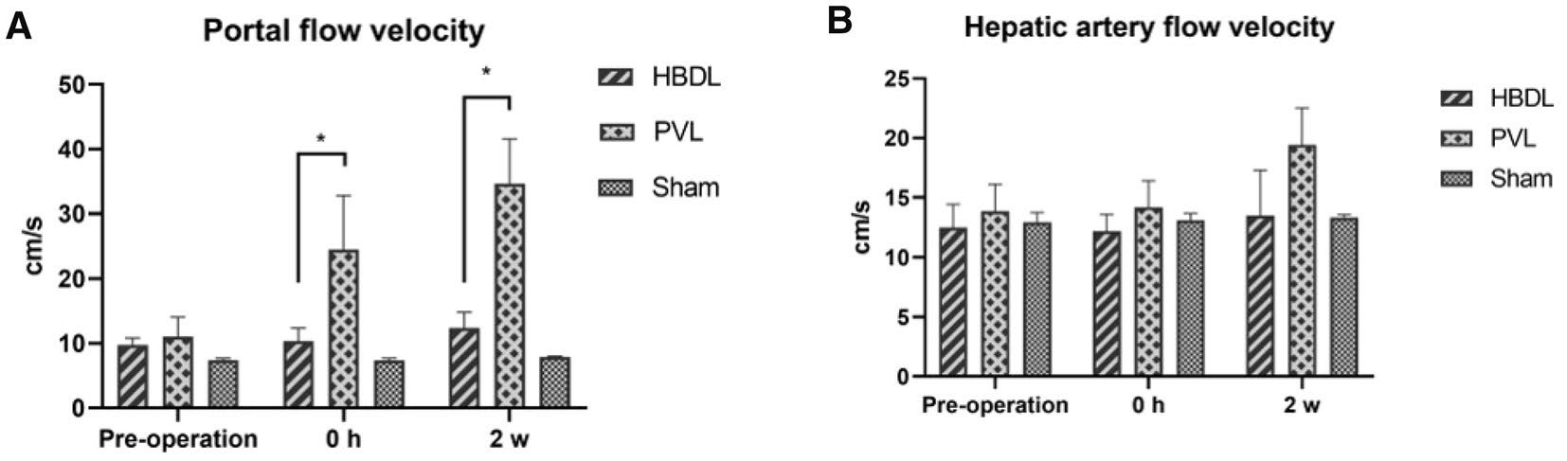
Changes in portal vein flow rate (**A**) and arterial flow rate (**B**) in the right lateral lobe of the liver in the three groups of rabbits at different time points.

PVL can cause changes in hemodynamics, and so changes in blood flow were measured before and after surgery in each group of rabbits. Compared with that before the operation, there was no significant change in portal blood flow velocity in the right lateral lobe in the HBDL group immediately after the operation and two weeks after the operation. In contrast, the portal blood flow velocity of the right lateral lobe in the PVL group increased significantly after surgery, and it remained increased until two weeks after surgery, which was significantly different from that of the HBDL group (*P* < 0.05). However, the portal diameter did not change significantly between the two groups during the two weeks after the operation. These results showed that the portal blood flow in the PVL group was significantly higher than that in the HBDL group. Compared with that in the sham group, the arterial blood flow velocity of the right lateral lobe in the PVL group increased after ligation, but the difference was not statistically significant (*P* > 0.05) (Fig. 7).

In the mouse model, the levels of IL-6 and TNF-α in the HBDL group were significantly higher than those in the sham group the first week after the operation (*P* < 0.05) (Supplementary Fig. S6).

### Histopathological examination

**Figure 8:**
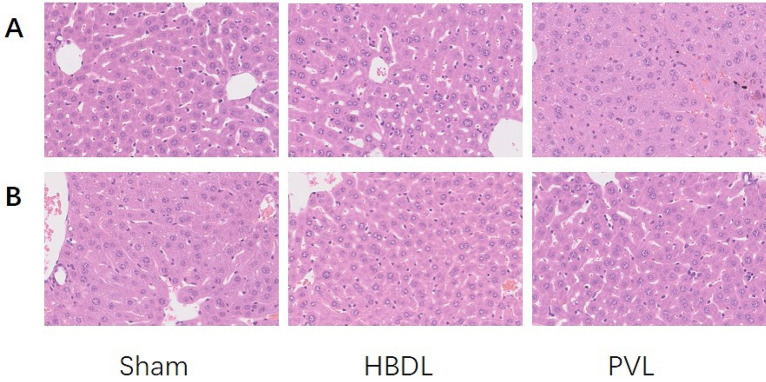
Observe the general liver histological changes caused by hilar biliary ligation or portal vein ligation by HE staining. The first day after operation (**A**); the first week after operation (**B**). (× 400).

The first day after the operation, although mild hepatic cord structure disorder and hepatocyte edema appeared in the unligated right lateral lobe of liver in both HBDL group and PVL group, the narrowing of the sinusoidal space and congestion in PVL group were more common, and occasionally multifocal necrosis of liver lobules was seen in the first week after surgery, the histology of the two groups was generally normal (Fig. 8).

We compared the liver function, the expression of genes and histological changes of the HBDL group and the PH group of mice. It is worth noting that compared with the PH group, the TBA level of the HBDL group was significantly higher. For detailed data, please refer to the Supplementary Information file. (Supplementary Figs. S7, S8, S9 and S10).

## Discussion

In this study, the unligated hepatic lobe showed a significant proliferative response in the animal model of hilar bile duct ligation. Liver volume or liver weight is the most objective indicator that directly reflects liver regeneration. After hilar biliary tract ligation, the volume and weight of the unligated liver lobe increased significantly. In the rabbit model, the unligated right lateral lobe liver volume increased by 119.4%, and the weight increased by 132.1% in the HBDL group at 2 weeks after the operation. In addition, the volume and weight of the right lateral lobe increased most significantly within the first week after surgery. We also observed in the mouse model that the liver proliferation was most significant in the first week after the operation. The proliferation rate of the hepatic lobe in the PVL group was comparable to that of the HBDL group. Chronic proliferative cholangitis caused by stones can cause obstructed lobe atrophy and hypertrophy of the other lobes, but this process takes a long time, the effect is not specific, and there are many complications, such as bacterial infection^27^. Therefore, the model we established is a rapid hepatic hyperplasia model. PCNA is an auxiliary protein required for DNA synthesis in eukaryotic cells and is directly involved in DNA replication during cell proliferation. Changes in the expression level of PCNA are consistent with the occurrence of DNA synthesis, reflecting cell proliferation^28^. The rate of positive PCNA staining in the HBDL group was significantly higher than that in the sham operation group, suggesting that liver hyperplasia in the animal model was clear. AST and ALT are the main indexes of liver injury. of the degree of damage to liver function may affect liver regeneration^29^. Compared with that in the PVL group, liver damage in the HBDL group was not exacerbated, and both groups showed transient liver function damage. Changes in bilirubin and albumin levels also illustrate this point. Studies have shown that liver resection can cause more serious liver damage than portal ligation^30,31^. Compared with the intrahepatic vascular system, bile duct ligation in a specific hepatic lobe can avoid massive hemorrhage caused by vascular surgical injury, which may be safer and more accurate^32^. Therefore, the animal model established in this study is a safe and effective animal model for the study of liver hyperplasia and does not cause serious damage to liver function to avoid impact on liver hyperplasia.

In both the partial hepatectomy and PVL-induced liver hyperplasia models, proliferation is affected by many factors. Due to damage induce by hepatic parenchyma transection, changes in hemodynamic factors and increases in energy demand per unit liver volume occurred, and a series of signals and pathways were activated, which coordinated the start and end of compensatory growth in the remaining parenchyma. In fact, partial resection of the liver and portal ligation significantly increased the portal vein blood flow of the remaining liver^33^ and increased the vascular shear stress of portal vein blood in the hepatic sinus, which further stimulated endothelial cells in the portal vein sinus and induced substantial inflammation. These hemodynamic changes are accompanied by the rapid remodeling of the extracellular matrix (ECM) via the protease cascade reaction^34^, promote the release and activation of growth factors such as hepatocyte growth factor (HGF)^35^, and trigger liver regeneration, thus initiating the transcription of genes related to liver regeneration^36^. Enhanced portal blood flow also increases the utilization of metabolites and other molecules in the hepatocytes in the remaining liver tissue^4^. Animal models of partial hepatectomy or PVL are associated with many liver hyperplasia mechanisms, and various factors interact with each other, increasing the complexity of studying liver hyperplasia induced by bile acid in hepatic and intestinal circulation and the susceptibility to other unrelated factors. In this study, there were no significant hemodynamic changes in the HBDL group after the operation, while the portal vein velocity in the PVL group was significantly increased, resulting in a continuous state of portal hyperperfusion in the hepatic lobe. This finding also suggested that the HBDL group did not exhibit the effects of hemodynamic changes on hepatocyte proliferation.

We hypothesized that proliferation in the unligated liver lobes in the HBDL group was mainly due to bile acid. Bile acid is only synthesized in the liver and is the main component of cholesterol catabolism. The most important pathway of bile acid synthesis, which accounts for 90% of total bile acid synthesis, is controlled by CYP7A1^37,38^. Bile acid levels in plasma and the liver increased rapidly after PH or biliary ligation, and large amounts of BA spread throughout the organism (systemic BA overload) through the blood circulation^7^. The rest of the liver has an adaptive response to prevent liver damage and allow normal regeneration^6^. An experiment in rats confirmed that regeneration of the liver decreased significantly when bile acid pools were emptied before PH, and this effect was reversed after an increase in duodenal bile acid^13^. BA and its receptors constitute a signaling network that is not well understood in the context of liver proliferation and the prevention of liver cell injury^39^. The basic principle of the rabbit and mouse HBDL models established in this study is to increase the level of bile acid through hilar ligation of certain bile ducts to evaluate the potential mechanism by which bile acid affects liver regeneration. The bile acid level in the rabbit model increased significantly the first day after the operation, reached a peak at the first week after the operation, which was consistent with the increase in liver volume, and was significantly different compared with those of the other two groups. We verified in a mouse model that bile acid levels increased significantly the first week after surgery. BA may mediate the hepatoproliferative response induced by the stimulation of farnesoid X receptor (FXR)^40,41^. FXR is a nuclear receptor that recognizes bile acids as endogenous ligands and is highly expressed in liver and intestinal epithelial cells^10^. In FXR-deficient mice, liver regeneration was damaged after PH^5^. After BA bound to FXR, the expression of small heterodimer partner (SHP) was induced, which inhibited BA synthesis by downregulating the expression of CYP7A1 and steroid 12α-hydroxylase (CYP8B1)^42,43^. In the liver, FXR seems to directly promote hepatocyte proliferation by inducing the transcription factor FoxM1b, which is necessary for hepatocyte proliferation^44^. The FXR-dependent adaptive response occurs in not only hepatocytes but also intestinal epithelial cells during liver regeneration. Intestinal FXR induces the expression of fibroblast growth factor 15 (FGF15 in mice, FGF19 in humans); FGF15/19 is secreted and reaches the liver through the portal circulation and then inhibits CYP7A1 expression and bile acid synthesis through signal transduction mediated by FGFR4 and β-Klotho^16^. FGF15/19 may also contribute to liver regeneration by upregulating proliferation-associated genes such as FoxM1b^45,46^. Therefore, we verified the gene expression of bile acid-related receptors in liver tissue and terminal ileum tissue in a mouse model. The expression levels of FXR, FGF15 and FoxM1b in the HBDL group were significantly higher than those in the sham group, and the expression of CYP7A1 was decreased. This finding was suggested that proliferation in the HBDL group was mainly due to bile acid and its pathway. The animal model of hilar bile duct ligation did not exhibit liver parenchymal transection injury, significant hemodynamic changes or systemic stress-related inflammation. In addition, cholestatic injury in an animal model of high ligation of the biliary tract occurred only after the ligation of the liver lobe, without a systemic effect^47^, and the interference factors were relatively small. Therefore, this model has high relevance in the study of bile acid enterohepatic circulation-induced liver hyperplasia.

In addition, most of the previously established animal models of bile acid-induced liver regeneration were partial hepatectomized mice. However, rabbits are a good animal model for studying the metabolism of human cholesterol and bile acid and are widely used in the study of blood lipids^48^. Compared with that of mice, rabbit anatomical structure and bile acid and cholesterol metabolism sequences have increased similarity with those of humans^49^. Moreover, the rabbit model has more advantages in observing the continuity of liver regeneration. Therefore, this study established a HBDL rabbit model and confirmed this model could be used to study bile acid-induced liver regeneration, filling the blank in this area.

This study also has value for further research. Interleukin-6 (IL-6) is considered to be the key cytokine associated with liver regeneration and is the main activator of signal transduction and STAT3. After partial hepatectomy, TNF-α induces the production of IL-6 by enhancing NF-κB, which then induces the expression of IL-6. IL-6 is an essential substance that causes abnormal liver proliferation^31,50^. The levels of TNF-α and IL-6 in mice were significantly higher than those in the sham group the first week after the operation. We hypothesize that hilar bile duct ligation may cause inflammation in the liver lobe, which in turn leads to the production of cytokines. Cytokines may also act on liver proliferation. The relationship between bile acids and cytokines needs further exploration. In addition, we only examined some of the key nodes in the bile acid pathway, and other receptors, such as TGR5, are also related to bile acid metabolism and liver hyperplasia^51^; thus, more comprehensive verification is needed in a variety of animals.

Several limitations need to be considered when interpreting the current study. First of all, in this study, we did not use an animal model of PH as a control group. Previous experiments have indicated that without other interventions, due to excessive inflammation and high blood flow perfusion, animals die quickly within 24 h with 90% hepatectomy model in rats in which only the caudate lobe of the liver was retained., and residual hepatic regeneration cannot be initiated^30,52^. In consequence, we adopted the sham group or PVL group as the control group in order to improve the stability of the animal model. In addition, FXR-deficient and other knockout animals were not used for modeling. Moreover, the testing of cytokines is not comprehensive, such as the level of TGF-β and HGF. Therefore, this animal model does not completely exclude the influence of inflammatory factors.

In conclusion, the HBDL model is simple to establish and exhibits good surgical tolerance. The model has definite proliferative effect and strong specificity of bile acid pathway. This is an ideal animal model to study the mechanism of liver regeneration through bile acid pathway.

## Supplementary Information

Supplementary Information.
