# Supplementary material for: A modified animal model of hepatic regeneration induced by hilar bile duct ligation
Source: Sci Rep. 2021 Oct 12;11:20201. doi: 10.1038/s41598-021-99758-z (PMC8511257; doi:10.1038/s41598-021-99758-z)
Supplement: Supplementary file 1 — Supplementary Information. [file 41598_2021_99758_MOESM1_ESM.docx]

**Supplementary Information**

**A modified animal model of hepatic regeneration induced by hilar bile duct ligation**

Tao Li^1^; Yichao Chai^2^; Pengkang Chang^1^; Fenggang Reng^1^; Zhao Xue^1^; Hongke Zhang^1^; Yi Lv^1^; Liangshuo Hu^1^

1 Department of Hepatobiliary Surgery and Institute of Advanced Surgical Technology and Engineering, The First Affiliated Hospital of Xi’an Jiaotong University, Xi’an, China.

2 Department of Oncology, The Second Affiliated Hospital of Xi'an Jiaotong University.

**Corresponding Author:**

Liangshuo Hu

huliangshuo1983@hotmail.com

*
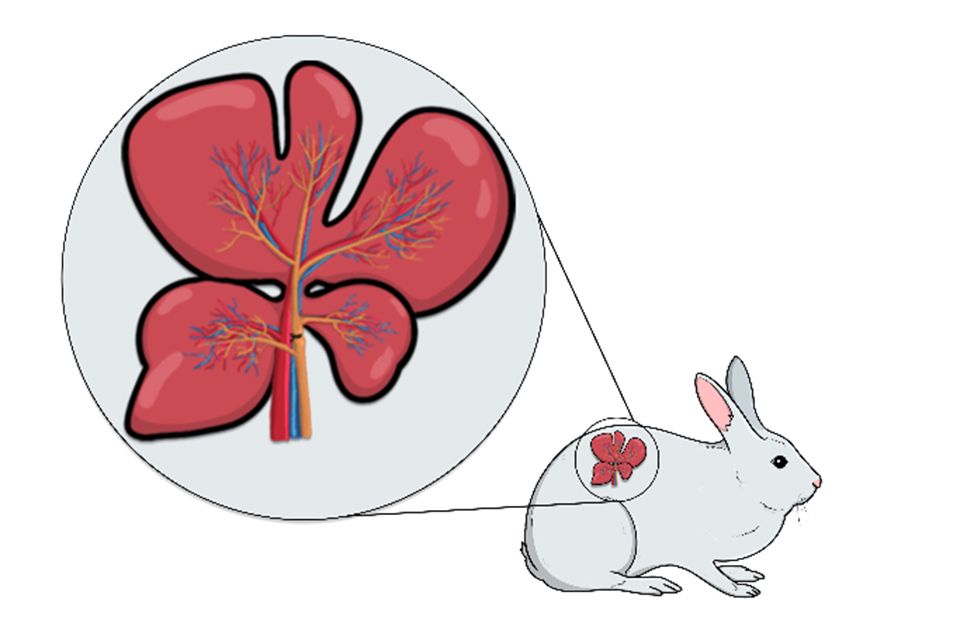
*
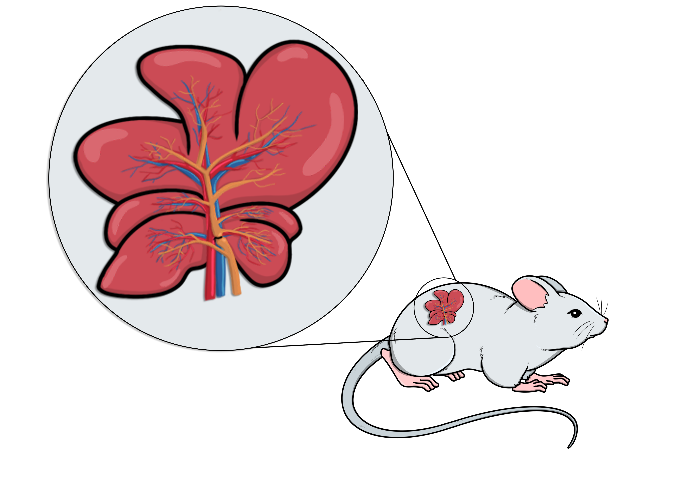
*Supplementary Figure S1: Animal model of hilar bile duct ligation*

*Supplementary Table S1*

| Operative techniques | Purpose | Distinction with HBDL model | Literatures |
| --- | --- | --- | --- |
| The bile duct and portal vein were ligated 90% simultaneously. | Observe the proliferation of liver lobe | In the HBDL model, the hilar bile duct was ligated alone, and the influence of hemodynamics on liver regeneration is excluded. | (Ren et al., 2015) |
| Common bile duct ligation | Evaluating cholangiocyte proliferation,  apoptosis and portal fibrosis due to extrahepatic cholestasis | The HBDL model does not cause serious liver injury and cholestasis. The tolerance of animals in HBDL model was good. | (Johnstone and Lee, 1976)  (Geerts et al., 2008)  (Tag et al., 2015)  (Aller et al., 2010) |
| Selective bile duct ligation | Evaluation of liver parenchymal fibrosis after cholestasis | The aim of HBDL model is bile acid-induced liver regeneration. The proportion and position of biliary ligation are different. | (Fickert et al., 2002)  (Tannuri et al., 2012) |
| Partial common bile duct ligation | Established for a model of acute cholestasis | The HBDL model does not cause serious liver injury and cholestasis. | (Heinrich et al., 2011) |

*Supplementary Table S2*

*Volume of the right lateral lobe of the liver in rabbits at different time points (n=18, ml, mean±SD)*

| Group | Preoperative | 1 week after the operation | 2 weeks after the operation |
| --- | --- | --- | --- |
| Sham | 12.74±1.58 | 14.53±1.18 | 13.18±1.88 |
| HBDL | 15.89±1.23 | 32.46±7.40 | 34.86±3.91 |
| PVL | 22.16±2.48 | 32.64±6.82 | 36.92±14.26 |

*Supplementary Table S3*

*Weight of the right lateral lobe of the liver in rabbits at different time points (n=18, g, mean±SD)*

| Group | Preoperative | 1 week after the operation | 2 weeks after the operation |
| --- | --- | --- | --- |
| Sham | 12.71±2.01 | 16.92±1.54 | 17.77±3.91 |
| HBDL | 16.89±1.24 | 35.12±7.10 | 39.21±3.72 |
| PVL | 23.25±3.14 | 34.67±10.51 | 42.43±15.11 |


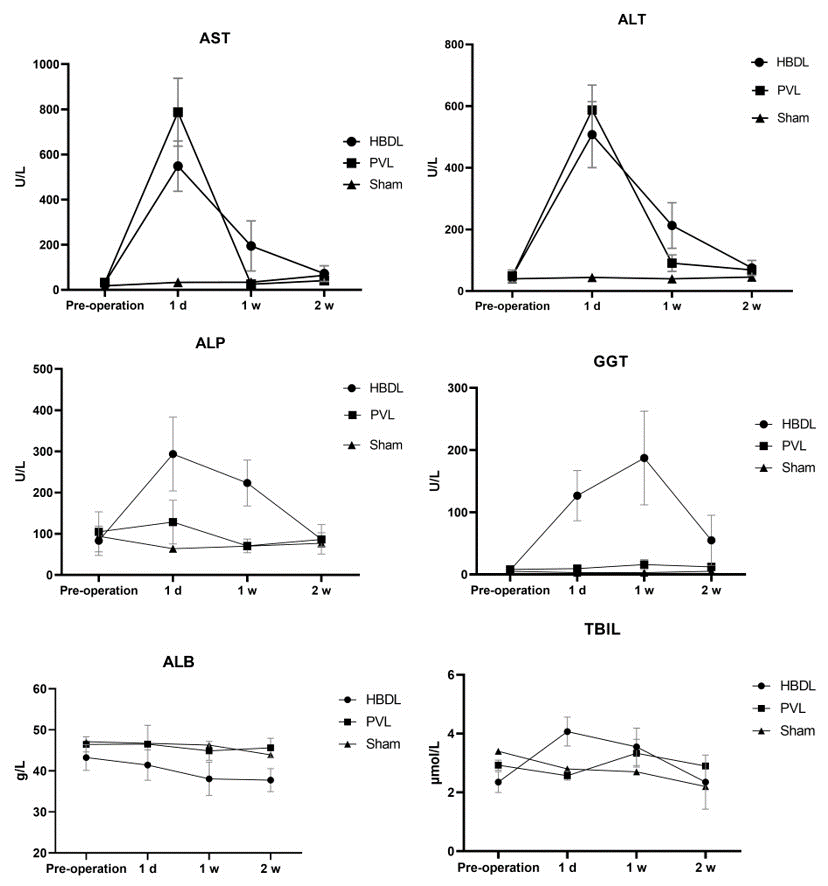


*Supplementary Figure S2: Serum levels of AST, ALT, ALP, GGT, ALB and bilirubin in the three groups of rabbits at different time points.*


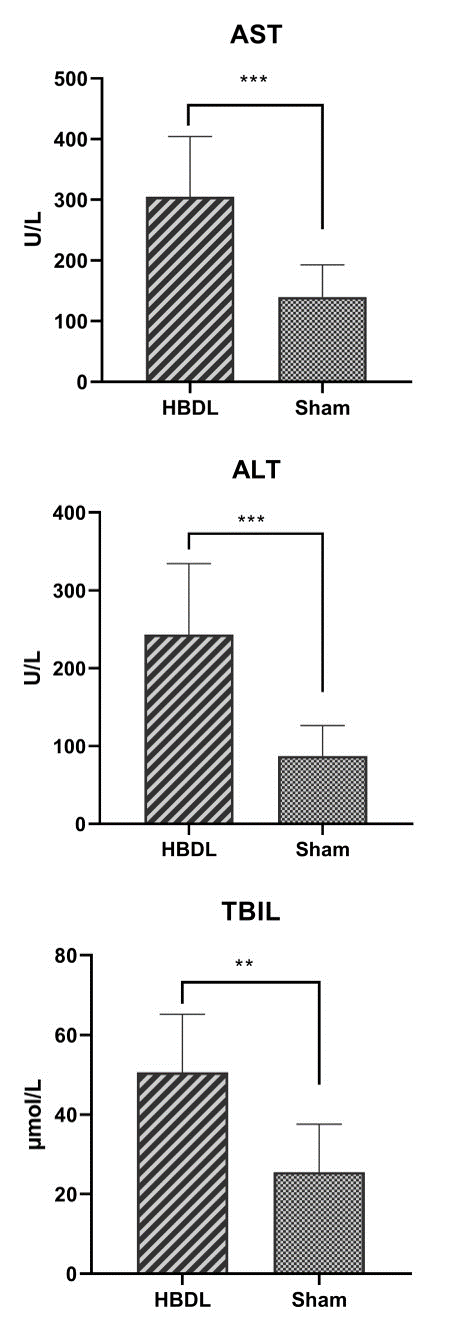


*Supplementary Figure S3: Serum levels of AST, ALT and bilirubin in HBDL mice the first week after surgery, *p<0.05 versus the sham group.*

*Supplementary Table S4 The positive rate of PCNA of different group at different time points（%， n=18， mean±SD)*

| Group | The positive rate of PCNA | |
| --- | --- | --- |
|  | 1 week | 2 weeks |
| Sham | 10±2.6 | 14±3.7 |
| HBDL | 55.4±7.5* | 33.4±5.6* |
| PVL | 51.6±6.3* | 37±4.9* |
| P | <0.05 | <0.05 |

*Compared with Sham group, *P < 0.05*


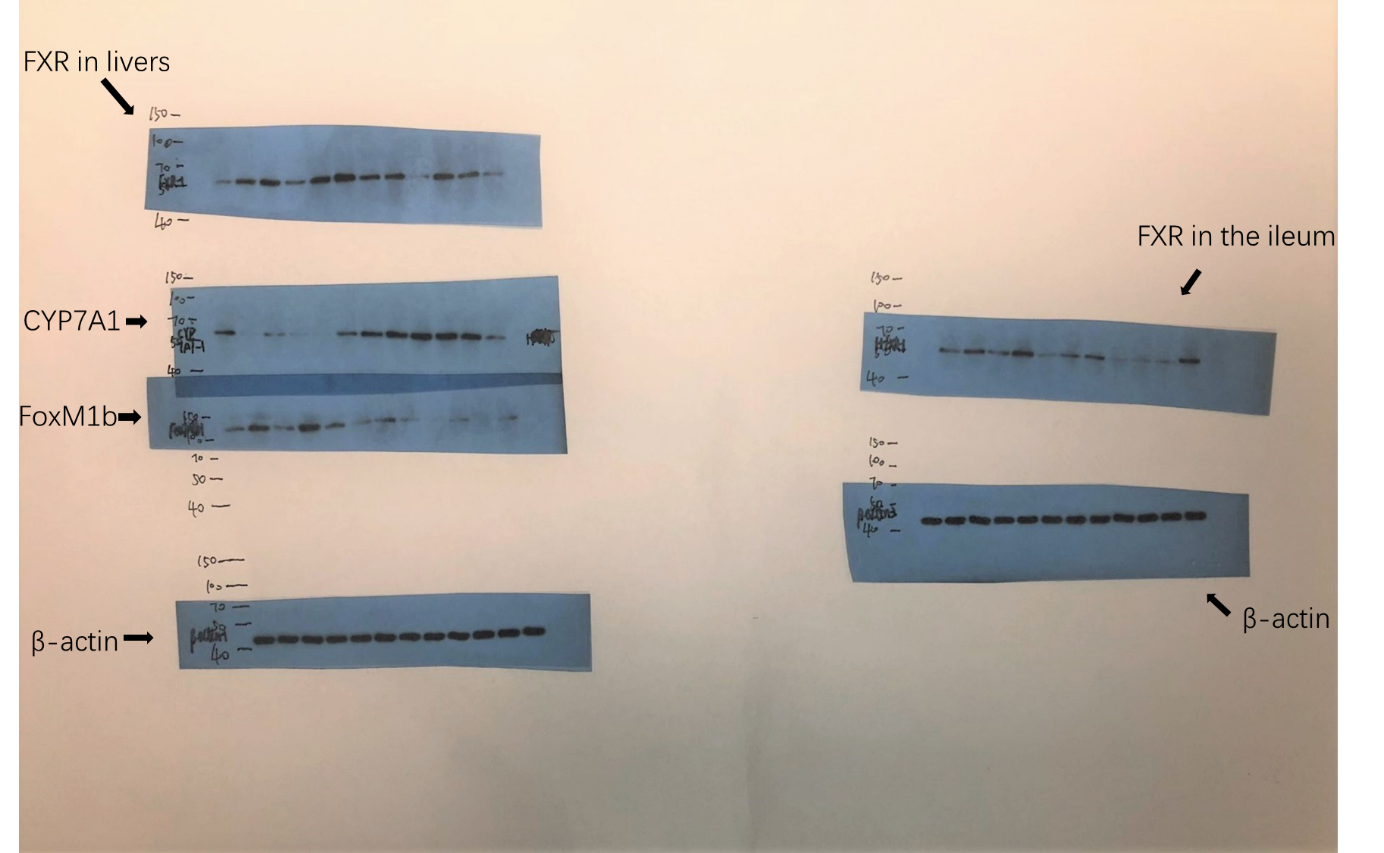


*Supplementary Figure S4: The full-length blots/gels including the key data presented in Fig. 5A. Representative blots of HBDL (left side of membranes) and Sham (right side of membranes) at the first day after the operation is indicated.*

*Note: The six blots on the left side of the membranes represent the HBDL group. The six blots on the right side of the membranes represent the Sham group.* *For FXR in the ileum, the five blots on the left side of the membranes represent the HBDL group. The five blots on the right side of the membranes represent the Sham group.*

*
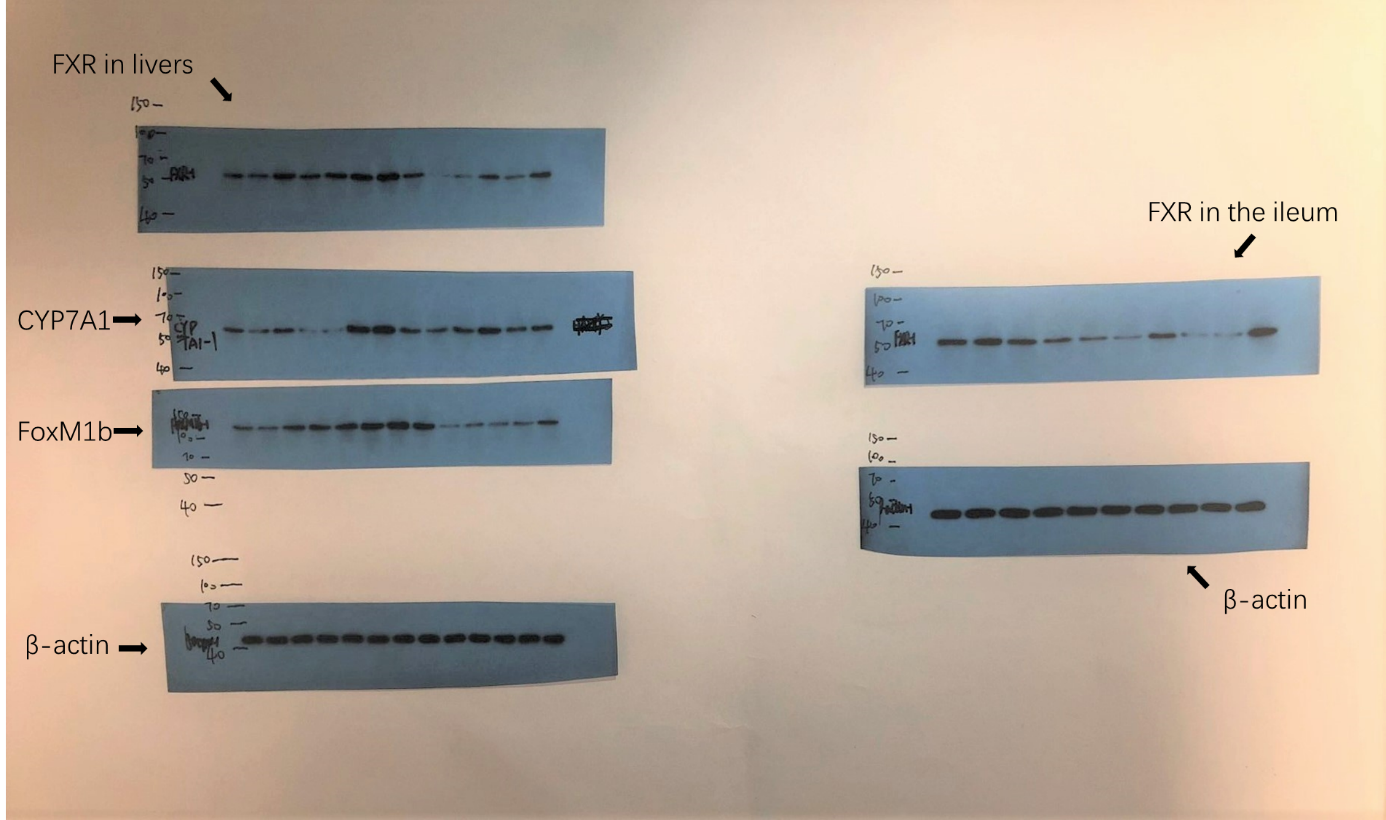
*

*Supplementary Figure S5: The full-length blots/gels including the key data presented in Fig. 5B. Representative blots of HBDL (left side of membranes) and Sham (right side of membranes) at the first week after the operation is indicated.*

*Note: The seven blots on the left side of the membranes represent the HBDL group. The six blots on the right side of the membranes represent the Sham group.* *For FXR in the ileum, the five blots on the left side of the membranes represent the HBDL group. The five blots on the right side of the membranes represent the Sham group.*

*
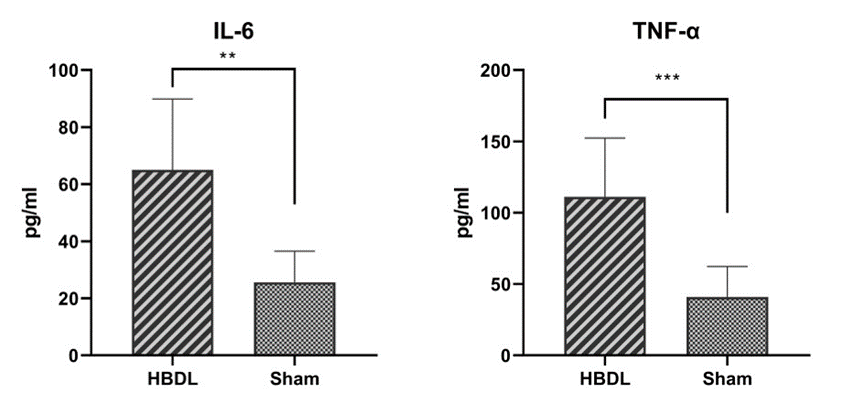
*

*Supplementary Figure S6: Serum IL-6 and TNF-α levels in HBDL mice the first week after surgery, *p<0.05 versus the sham group.*


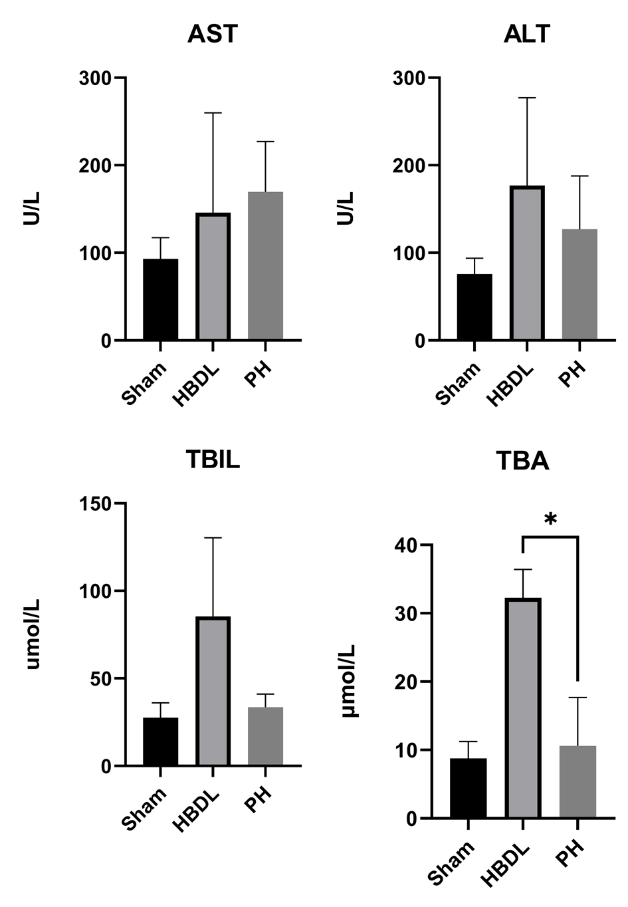


*Supplementary Figure S7: Serum levels of AST, ALT, TBIL and TBA in HBDL mice the first week after surgery.*


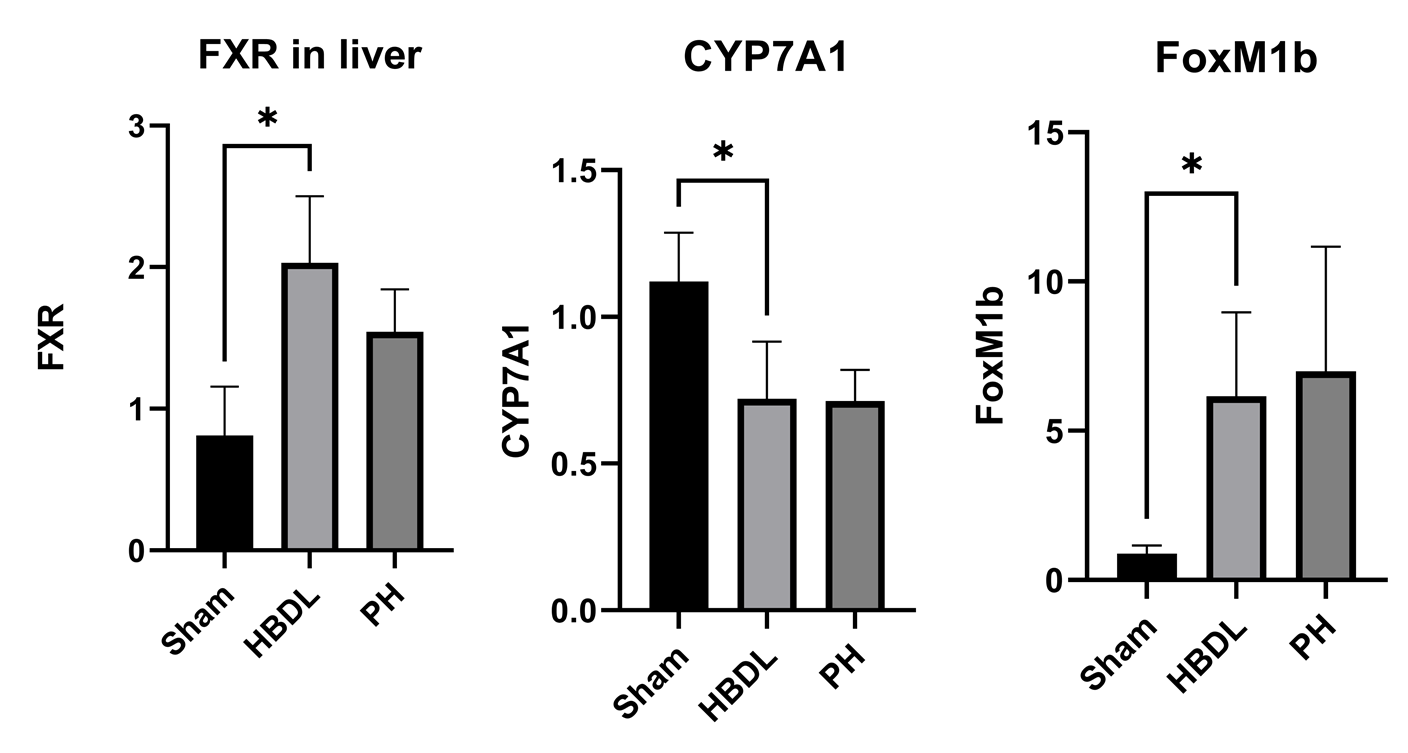


*Supplementary Figure S8: Expression of FXR in the livers, CYP7A1 in the livers and FoxM1b in the livers of HBDL mice the first week after surgery.*


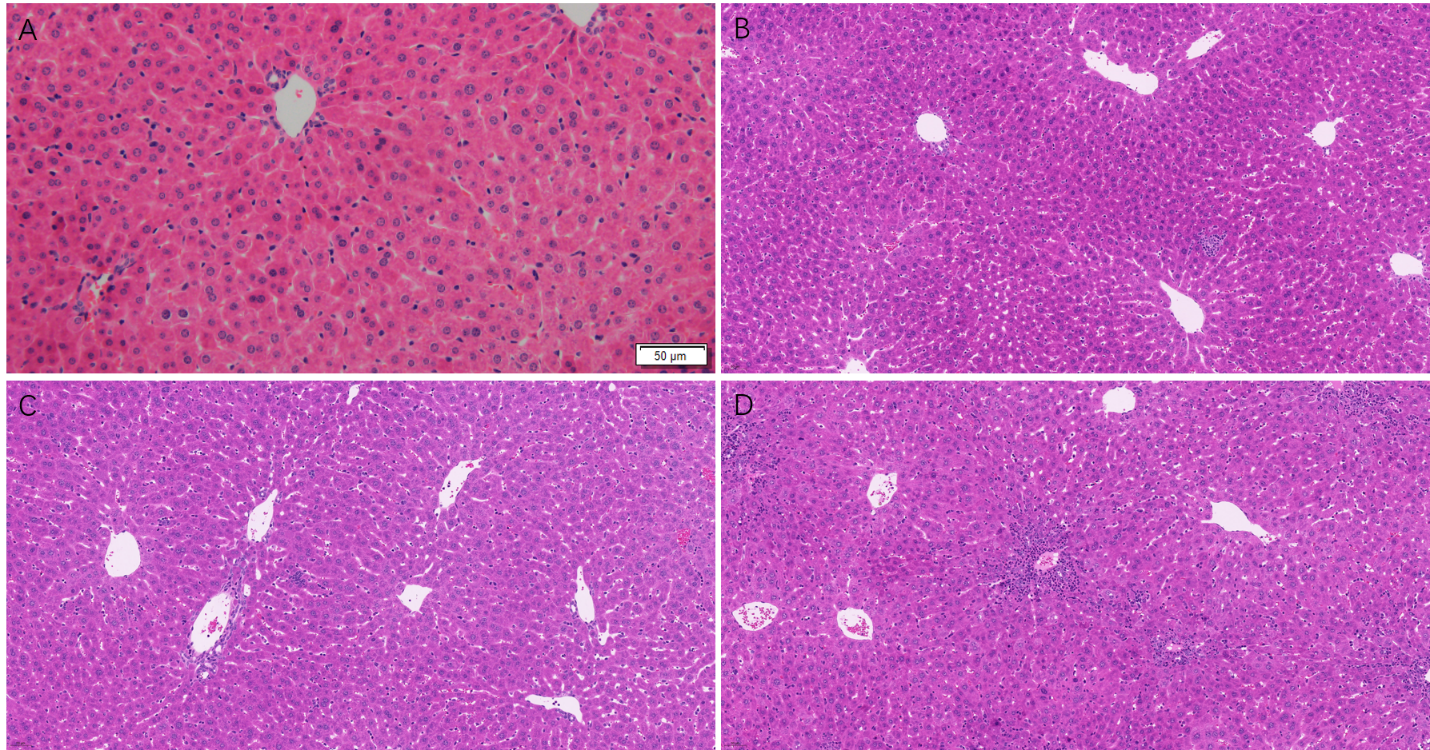


*Supplementary Figure S9: Observe the general liver histological changes caused by hilar biliary ligation or PH by HE staining. Sham group (A); PH group (B)；Unligated liver lobe of HBDL group（C）；Ligated liver lobe of HBDL group（D）. (×200)*


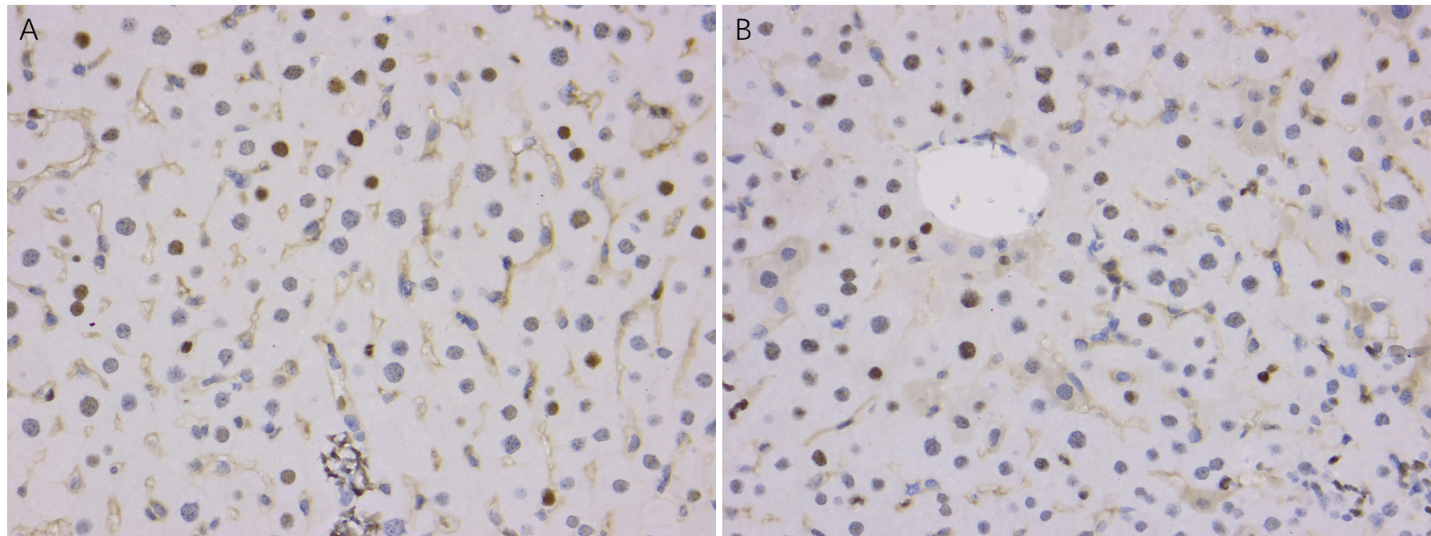


*Supplementary Figure S10: DNA synthesis in hepatocytes was assessed at different time points by immunohistochemical evaluation of PCNA incorporation into DNA. HBDL group (A); PH group (B). (×400)*
